# Effectiveness of Educational Interventions for Health Workers on Antibiotic Prescribing in Outpatient Settings in China: A Systematic Review and Meta-Analysis

**DOI:** 10.3390/antibiotics11060791

**Published:** 2022-06-10

**Authors:** Kunhua Zheng, Ying Xie, Lintao Dan, Meixian Mao, Jie Chen, Ran Li, Xuanding Wang, Therese Hesketh

**Affiliations:** 1 People’s Hospital of Kaihua, Quzhou 324300, China; kunhuazheng@sina.com (K.Z.); mmx84089@163.com (M.M.); 2 Center for Global Health, Zhejiang University School of Medicine, Hangzhou 310000, China; xieying1110@zju.edu.cn (Y.X.); lintaodanmedicine@zju.edu.cn (L.D.); med_chenjie@zju.edu.cn (J.C.); ranli38@zju.edu.cn (R.L.); t.hesketh@zju.edu.cn (T.H.); 3 Institute for Global Health, University College London, London WC1E 6BT, UK; 4 Department of Antimicrobial Stewardship, The Second Affiliated Hospital of Zhejiang University School of Medicine, Hangzhou 310000, China

**Keywords:** antibiotics, stewardship, resistance, educational intervention, outpatient, China

## Abstract

Educational interventions are considered an important component of antibiotic stewardship, but their effect has not been systematically evaluated in outpatient settings in China. This research aims to evaluate the effectiveness of educational interventions for health workers on antibiotic prescribing rates in Chinese outpatient settings. Eight databases were searched for relevant randomized clinical trials, non-randomized trials, controlled before–after studies and interrupted time-series studies from January 2001 to July 2021. A total of 16 studies were included in the systematic review and 12 in the meta-analysis. The results showed that educational interventions overall reduced the antibiotic prescription rate significantly (relative risk, RR 0.72, 95% confidence interval, CI 0.61 to 0.84). Subgroup analysis demonstrated that certain features of education interventions had a significant effect on antibiotic prescription rate reduction: (1) combined with compulsory administrative regulations (RR With: 0.65 vs. Without: 0.78); (2) combined with financial incentives (RR With: 0.51 vs. Without: 0.77). Educational interventions can also significantly reduce antibiotic injection rates (RR 0.83, 95% CI 0.74 to 0.94) and the inappropriate use of antibiotics (RR 0.61, 95% CI 0.51 to 0.73). The limited number of high-quality studies limits the validity and reliability of the results. More high-quality educational interventions targeting the reduction of antibiotic prescribing rates are needed.

## 1. Introduction

Antimicrobial resistance (AMR) is one of the greatest challenges threatening global health, economy, and security today. It is estimated that by 2050, AMR will be responsible for 10 million deaths per year [1]. The World Bank has calculated that the healthcare costs of AMR will be as high as one trillion US dollars per year by 2050 [2]. Urgent action is needed to address this worsening situation [3].

China is the largest consumer of antibiotics across the medical and agricultural sectors: around half of the total 162,000 tons is used in medicine [4]. Concerns about the impact of massive antibiotic use on AMR have led the Chinese government to introduce a series of regulations on antibiotic use with a focus on rational prescribing in clinical practice [5]. Since 2011, several announcements relating to antimicrobial stewardship have been published by the National Health Commission. In 2016, the National Health Commission, together with 13 other ministries, jointly issued the National Action Plan to Contain Antibacterial Resistance (2016–2020) [6]. It emphasizes the need to strengthen the rational use of antimicrobial drugs and provide continuing education for medical staff on an annual basis with exams that need to be passed to continue prescribing antibiotics [6]. In China, doctors can be qualified to prescribe antimicrobial drugs after training and assessment. They can prescribe different levels of antimicrobial drugs according to their professional positions. The prescription behaviors of prescribers is also not independent, as some extrinsic factors such as other healthcare givers, patients and health systems may affect the antibiotic prescription as well [7].

Despite attempts to find alternative types of medication to cost-effectively treat those infections for which antibiotics are commonly used [8], interventions to improve antibiotic prescribing behavior may be more direct and effective. Globally, around 90% of antibiotic prescriptions in medicine are prescribed in outpatient settings, where it is well known that antibiotic overuse and misuse are common [9,10]. In China, a nationwide study collected data for antibiotics prescribed at 14,736,483 out-patient visits and found that only 16% were appropriately prescribed, 31% potentially appropriately prescribed and 48% inappropriately [11]. The optimization of antibiotic use in the outpatient setting has been increasingly perceived as a chance to improve patient safety [10]. The rational use of antibiotics, aligned with evidence-based recommendations, can reduce patients’ unnecessary exposure to the side effects of antibiotics.

In general, antimicrobial stewardship programs have the following core goals: (1) appropriate empiric antibiotic selection appropriately dosed-based on organ function; (2) de-escalation upon receipt of culture and sensitivity; (3) conversion from intravenous infusion to oral dose forms; and (4) appropriate duration of therapy. Previous studies have explored various types of interventions to improve antimicrobial stewardship [12]. Educational interventions are considered an effective and vital component of antimicrobial stewardship [13]. A global systematic review of 78 educational interventions in 2014 showed improved adherence to guidelines in 46% of included studies, and reduced antibiotic prescribing in 41% of studies [14]. A more recent systematic review and meta-analysis worldwide showed that digital education was more effective and cost-effective than traditional education methods in improving rational prescribing [15]. The need for better education on AMS with feedback on prescribing choices has been acknowledged by Chinese doctors [16]. However, such education programs are much more common in Europe and North America than in countries where antibiotic misuse is a serious problem [17]. However, despite the requirements for doctors in China to undergo specific training in AMS, there is little published evidence for the effectiveness of such programs.

This systematic review with meta-analysis was conducted to determine the effectiveness of educational interventions to improve rational antibiotic prescribing in outpatient departments in hospitals and clinics in China. This study aims to: (i) explore the effectiveness of educational interventions on the antibiotic prescribing rate and rational antibiotic use; (ii) compare the influence of educational interventions combined with or without feedback, compulsory administrative regulations, and financial incentives on antibiotic prescription rates.

## 2. Methods

The protocol of the study was registered in PROSPERO (CRD42021227517). The search followed the Preferred Reporting Items for Systematic reviews and Meta-Analyses (PRISMA) protocol, for the transparent reporting of systematic reviews and meta-analyses (checklist in Table S1).

### 2.1. Search Strategy

We searched the following databases from January 2001 to July 2021: PubMed, Web of Science, Embase, Ovid Medline, Cochrane Library, China National Knowledge Infrastructure (CNKI), China Science and Technology Journal Database and Wanfang Database. Relevant studies published in English and Chinese were included and different search strategies were applied. The search strategies were designed according to the PICOS, details can be seen in Table S2.

JC and YX were responsible for screening and identifying relevant studies. YX and LD worked independently screening titles and abstracts of studies. Disagreements were referred to a third researcher (JC) or resolved through discussion with all authors.

### 2.2. Inclusion and Exclusion Criteria

We referred to the PICOS (Participants, Interventions, Comparisons, Outcomes, and Study designs) framework for inclusion and exclusion criteria. We excluded studies conducted outside China or papers published as reviews, protocols, or conference abstracts.

#### 2.2.1. Participants

Doctors are the prescribers at outpatient facilities, who were deemed as the primary participants in our study. We also included studies targeting both prescribers and other health workers such as caregivers, nursing staff and pharmacist. Though these personnel did not prescribe drugs directly, they may affect the prescribing behaviors of doctors to some extent.

#### 2.2.2. Interventions

We included studies comprising educational interventions as individual programs or incorporated with other interventions. An educational intervention was defined as a program designed to encourage doctors to improve their practice performance through information or training strategies. Specifically, it includes delivery of print/audio-visual learning materials (printed matter, protocols, guidelines, self-instruction materials or manual) or interactive group learning or discussion (lectures, seminars, conferences, group sessions or tutorials) [14]. Non-educational interventions include: (1) prescription feedback (consisting of peer or expert review of prescriptions and feedback); (2) mandatory administrative regulations, including setting specific prescribing targets, implementing prescription audit and/or displaying ranking information); (3) financial incentives (reward or punishment).

#### 2.2.3. Comparisons and Outcomes

Comparisons were made with usual practice. The primary outcome was antibiotic prescription rate. Secondary outcomes were specific prescription rates for a particular disease, types of antibiotics prescribed, that is, broad-spectrum or parenteral antibiotics, prescription rates for 2 or more kinds of antibiotics, and knowledge improvement.

#### 2.2.4. Study Design

With reference to EPOC (Cochrane Effective Practice and Organization of Care), the following study designs were included [18]: randomized clinical trials (RCTs), non-randomized trials (NRCTs), controlled before-after studies (CBA) and interrupted time series (ITS) studies.

### 2.3. Data Extracting

We extracted the following items according to the Cochrane Handbook for Systematic Reviews: first author, year of publication, study design, setting, location China, participants, intervention details, target illness, duration and outcomes measures.

### 2.4. Risk of Bias Assessment

The risk of bias was assessed using EPOC recommended criteria for studies with a separate control group (RCTs, NRCTs and CBA, with 9 criteria) and ITS (with 7 criteria) [19]. Common criteria include missing outcome data, selection of reported outcomes, expected interventions and contamination. ITS additionally included assessments for predetermined and independent effect traits, while other designs included random sequences, assignments, baseline measurements and characteristics. The risk of bias is low if all criteria were scored as ‘low’; medium if one or two criteria were scored as ‘unclear’ or ‘high’, and high if three or more criteria were scored as ‘unclear’ or ‘high’ [12,20,21].

### 2.5. Data Analysis

A meta-analysis was performed on the primary outcome of antibiotic prescription rates, as well as secondary outcomes where enough studies were included. We extracted numbers of total prescriptions and prescriptions including antibiotics in both intervention and control groups pre- and post- intervention for RCTs or NRCTs. For CBA and ITS, we extracted numbers of total prescriptions and those where antibiotics were included pre- and post- intervention. Given the expected statistical heterogeneity, we estimated the pooled value with a random-effects model. The estimated effect size was shown in forest plots with risk ratio (RR) and 95% confidence intervals (95% CI).

Subgroup analysis was conducted in separated groups for education-only or education-plus interventions, with or without feedback interventions, compulsory administrative regulations, financial incentives and delivery of education online (learning materials, teaching or discussion via internet-, electronic-, and/or smart phone-based media) or offline (printed education materials, bulletin board in workplace or teaching or discussion face-to-face), to analyze the reduction of antibiotic prescription rate. To examine the heterogeneity, we applied a leave-one-out analysis. Cochrane’s Q statistic and I^2^ statistic, indicate the proportion of total variance due to heterogeneity. The possibility of publication bias was examined by Egger’s test, as well as funnel plots. EndNote X9 was used to store bibliography and Microsoft Excel was for data management. R software version 4.1.2 with Review Manager version 5.4 were used for analyses and plots making.

## 3. Results

### 3.1. Study Selection

**Figure 1:**
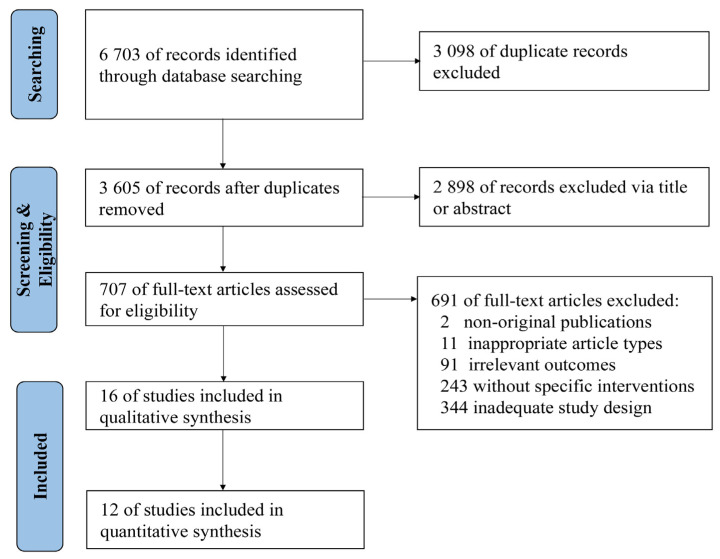
Flow diagram of study identification and screening.

A total of 6703 relevant studies were identified in the databases (Figure 1). After reviewing titles, abstracts, and full-text, 16 studies [22,23,24,25,26,27,28,29,30,31,32,33,34,35,36,37] were included in the qualitative synthesis and 12 [23,24,26,27,29,30,31,33,34,35,36,37] were included in the quantitative synthesis.

### 3.2. Study Characteristic

In the studies included (Table 1), seven studies [22,23,25,27,28,29,30] were in English and nine [24,26,31,32,33,34,35,36,37] were in Chinese. Only one study included all three levels of care (primary to tertiary hospitals) [33], four studies were conducted in secondary and/or tertiary hospitals [22,28,31,34] and 11 studies in primary care hospitals [23,24,25,26,27,29,30,32,35,36,37]. One study was conducted nationwide [22]; six in rural areas [23,24,26,29,30,35], and nine at the city level [25,27,28,31,32,33,34,36,37]. Study design included five cluster RCTs [23,27,29,30,33] (including two with different timings of follow-up [29,30], seven CBA studies [24,26,31,34,35,36,37], and four ITS studies [22,25,28,32]. The four ITS were followed-up from 11 to 48 months after the intervention and one cluster RCT was followed up for 12 months [29]. Other studies only obtained post-intervention data with no follow-up performed.

#### 3.2.1. Population

Many studies included caregivers, pharmacists and other medical staff, in addition to doctors, the prescribers. Six studies only targeted prescribers [25,26,27,29,30,32], while two studies included both prescribers and pharmacists [33,36] (without prescriptive authority in China), and another two studies included prescribers, pharmacists and caregivers as well [24,31]. Another six studies targeted all medical staff in outpatient settings [22,23,28,34,35,37], including two studies conducting universal interventions aimed to measure the implementation of an AMR regional policy [22,28], and the other four were set in primary care settings where other medical staff may easily influence doctors’ prescribing behaviors [23,34,35,37]. Only two studies reported actual numbers of participants, including 977 health workers in 100 township health centers [23] and 820 doctors in a tertiary hospital in Beijing [28]. The other 14 studies did not report the number of individuals included in the intervention, but rather analyzed the impact of the intervention on prescribing through hospital-wide antimicrobial prescribing.

#### 3.2.2. Intervention

Two studies consisted of education interventions only [23,33] and 14 were education-plus interventions (defined as interventions including measures other than education) [22,24,25,26,27,28,29,30,31,32,34,35,36,37]. Education interventions in the two studies used dissemination of learning materials such as text messages (containing recommendations for antibiotic management three times per week for 5 weeks) or brochures (for rational use of antibiotics), and organized lectures (concerning standards, rational use and management) [23,27]. In the studies with education-plus measures for the intervention group, nine used feedback of prescription or prescription patterns [25,26,27,28,29,30,31,32,36], six studies combined compulsory administrative regulations [22,26,27,31,34,35], six studies combined financial incentives or/and penalties [25,28,32,34,35,37], and one study introduced an automatic prescription screening system [28]. Intervention approaches included two studies which were conducted online [23,32], two combined online and offline [24,36], and 12 were conducted offline only.

Of the 16 papers, 15 analyzed the effectiveness of interventions versus no intervention, using a control group or comparison of pre- and post- intervention. Chen [23] compared the effect of online text messages (intervention group) and offline training lectures (control group) specifically for the treatment of upper respiratory tract infection.

#### 3.2.3. Outcomes of Interest

A total of 15 studies [22,23,24,25,26,27,28,29,30,31,32,33,34,35,36] reported antibiotic prescription rates, with 11 reporting specific changes in numbers of prescriptions of antibiotics [23,24,26,27,29,30,31,33,34,35,36], while the other four studies reported estimated monthly decline in antibiotics prescribing rates [22,25,28,32]. For secondary outcomes, seven studies reported parenteral use of antibiotics [27,29,30,34,35,36,37], four reported the inappropriateness rate of antibiotic prescription [26,35,36,37], and five reported use of multiple antibiotics [27,29,30,36,37]. Two studies investigated the changes in types of antibiotics pre- and post-intervention [26,37]. One also reported the top 10 prescription diagnoses using antibiotics from 2012 to 2014, where acute upper respiratory tract infections were the most common disease prescribed with antibiotics during the three years of follow-up [26]. One study reported the knowledge improvement and attitude change after interventions quantitively [23], while another reported qualitatively. One study measured the bacterial resistance rate and estimated its correlation to specific antibiotic prescription rates during the intervention implementation [28].

### 3.3. Risk of Bias Assessment

**Figure 2:**
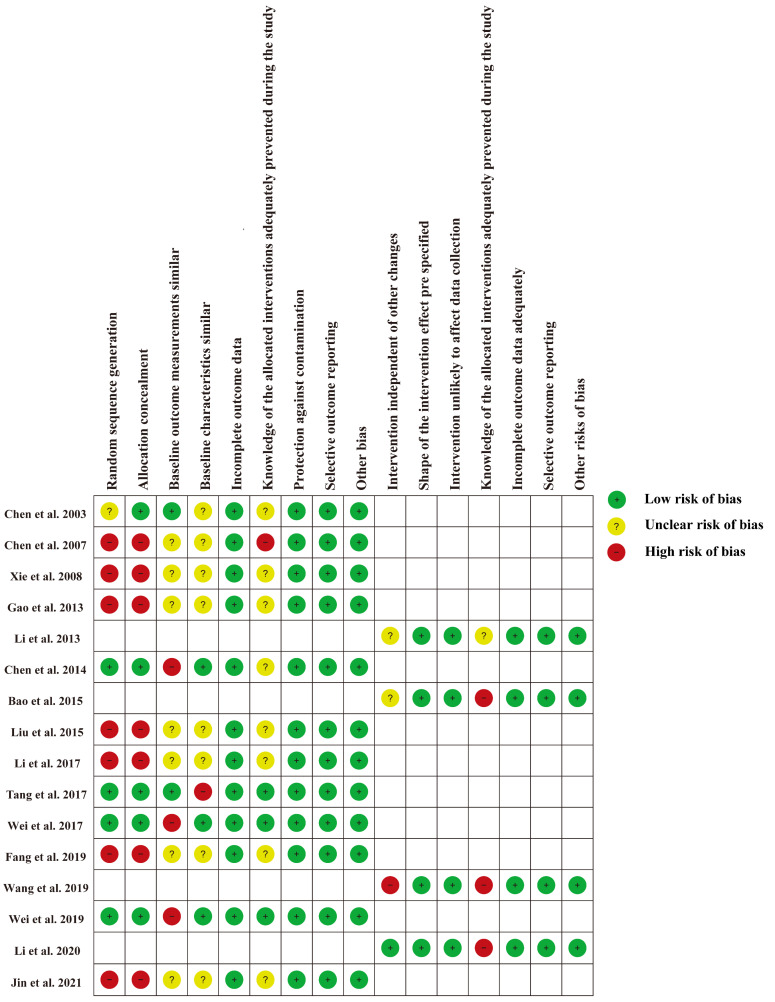
Risk of bias for each included study [22,23,24,25,26,27,28,29,30,31,32,33,34,35,36,37].

The risk of bias for each study is shown in Figure 2 and Figure S1. For 12 RCTs and CBA studies [23,24,26,27,29,30,31,33,34,35,36,37], the main risk bias derived from selection bias due to the nature of CBA studies [19], as well as potentially significant differences in expected outcomes at baseline. For four ITS studies [22,25,28,32], the main source of bias was that the interventions were not independent of other changes. Since educational interventions are delivered directly to doctors, all studies are not immune to lack of blinding of the allocated interventions.

### 3.4. Effects of the Interventions

#### 3.4.1. Antibiotic Prescription Rate

**Figure 3:**
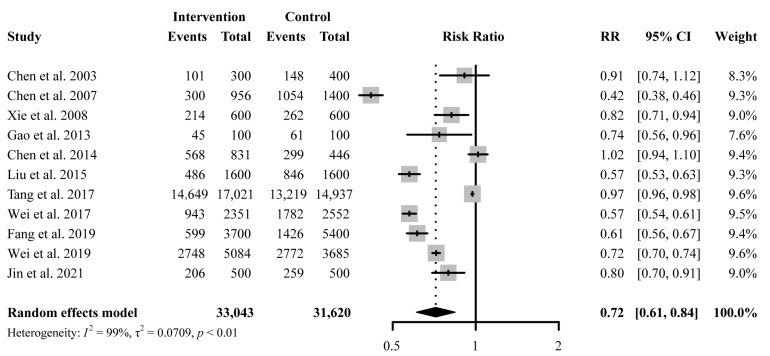
Effect of education-only intervention or plus other interventions on antibiotic prescription rate. Abbreviations: RR, risk ratio; CI, confidence interval [23,24,26,27,29,30,31,33,34,35,36].

**Figure 4:**
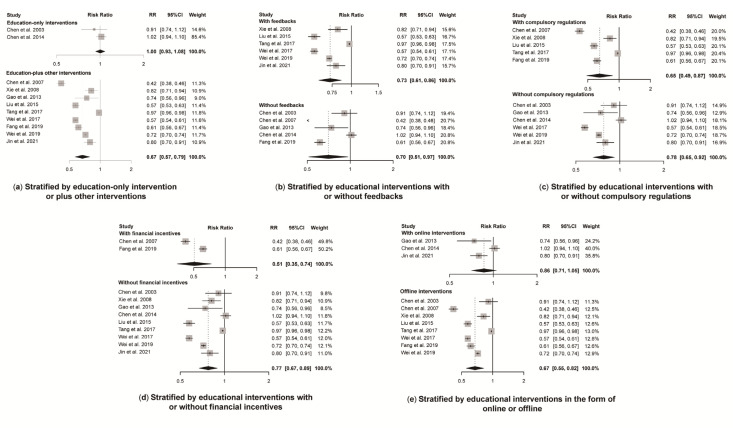
Effect of educational interventions with certain features on antibiotic prescription rate. Abbreviations: RR, risk ratio; CI, confidence interval. Notes: (**a**) Effect of education-only intervention or plus other interventions on antibiotic prescription rate; (**b**) Effect of educational interventions with or without feedbacks on antibiotic prescription rate; (**c**) Effect of educational interventions with or without compulsory regulations on antibiotic prescription rate; (**d**) Effect of educational interventions with or without financial incentives on antibiotic prescription rate; (**e**) Effect of educational interventions in the form of online or offline on antibiotic prescription rate [23,24,26,27,29,30,31,33,34,35,36].

A total of 11 studies reported antibiotic prescription rates with a total number of prescriptions. Educational interventions were found to reduce antibiotic prescription rates significantly (RR 0.72, 95% CI 0.61 to 0.84) (Figure 3). However, no difference was found in antibiotic prescription rate between intervention and control group for education-only interventions (RR 1.00, 95% CI 0.93 to 1.08), while education-plus interventions showed a significant reduction in reducing antibiotic prescription rates (RR 0.67, 95% CI 0.57 to 0.79) (Figure 4). Educational interventions with (RR 0.73, 95% CI 0.61 to 0.86) or without (RR 0.70, 95% CI 0.51 to 0.97) feedback interventions showed similar significant effects on antibiotic prescription rate reduction (Figure 4). In addition, interventions including compulsory administrative regulations (RR 0.65, 95% CI 0.49 to 0.87) reduced antibiotic prescription rates more than those without, as measured by pre-and post-introduction of compulsory regulations (RR 0.78, 95% CI 0.65 to 0.92) (Figure 4). Educational interventions combined with financial incentives (RR 0.51, 95% CI 0.35 to 0.74) also showed a greater effect on reducing antibiotic prescription rate than those without (RR 0.77, 95% CI 0.67 to 0.89) (Figure 4). There was no significant difference between intervention and control groups when receiving online interventions (RR 0.86, 95% CI 0.71 to 1.05), while offline interventions showed a significant difference (RR 0.67, 95% CI 0.55 to 0.82 (Figure 4). Given the heterogeneity in our main results, we also conducted a leave-one-out analysis, and none of the omitted studies significantly influenced our results (Figure S2).

#### 3.4.2. Parenteral Use of Antibiotics

Seven education-only or education-plus interventions showed pooled results comparing parenteral use of antibiotics in control and intervention groups. The intervention group had a 17% reduction in parenteral use rate (RR 0.83, 95% CI 0.74 to 0.94, details in Figure S3).

#### 3.4.3. Multiple Antibiotic Rates

The education-plus intervention did not reduce multiple antibiotic rates as reported in five studies (RR 0.73, 95% CI 0.72 to 1.29, Figure S4). Among those studies, two [36,37] reported on the use of two or three antibiotics respectively, while others reported an overall rate for two or more antibiotics.

#### 3.4.4. Antibiotic Prescription Inappropriateness Rate

Four studies documented antibiotic prescription inappropriateness using similar evaluation criteria in Table S3 except one [35] with unspecified criteria. Results in Figure S5 showed education-plus interventions did reduce antibiotic prescription inappropriateness rate (RR 0.61, 95% CI 0.51 to 0.73).

#### 3.4.5. Changes in Types of Antibiotics or Target Diseases

**Figure 5:**
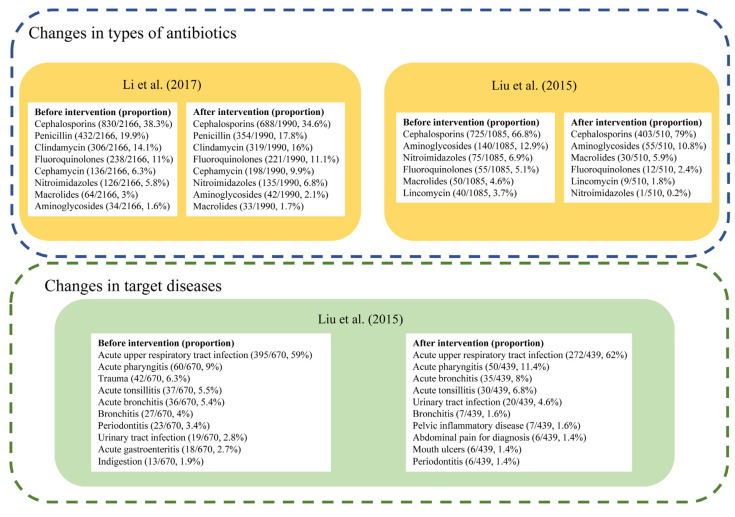
Changes of antibiotic prescription in types or target diseases [24,35].

Only two studies reported the specific types of antibiotics used (Figure 5). Both studies reported Cephalosporins as the most frequently used antibiotics before and after the intervention. In Liu’s study carried out in township health centers in Guangdong Province, cefuroxime tablets and ceftezole sodium for injection were the most widely used antibiotics in the included community health centers from 2012–2014 [26], and there was an increase in the proportion of Cephalosporins used from 66.8% to 79.0% following the intervention [26]. The other study by Li et al. reported a decrease from 38.3% to 34.6% of Cephalosporins in 2015, though it always ranked first, among 17 primary health centers in Zhejiang province [37].

Liu’s study also measured changes in antibiotic prescriptions for specific diseases [26]. While the antibiotic prescription rate showed an overall decline, the proportion of antibiotic prescriptions for acute upper respiratory tract infection increased from 46.7% to 56.0%, and numbers of antibiotic prescriptions and proportions for trauma, mouth ulcers, indigestion, acute gastroenteritis all declined [26].

#### 3.4.6. Knowledge Improvement

One cluster RCT compared the effect of sending learning text messages with traditional training workshops [23]. Researchers used 10 multiple-choice questions on the appropriate treatment of the selected diseases and complications via a telephone survey. The knowledge score increased 16% (95% CI: 15.7–16.3%) in the intervention group, with no significant changes in the control group. Another study reported qualitative results on doctors’ self-reported knowledge improvement. The participants reported better knowledge and improved confidence in the appropriate use of antibiotics and had increased their use of guidelines for prescribing following the intervention [29].

## 4. Discussion

Our study shows that education interventions can significantly reduce the antibiotic prescription rate when combined with other types of interventions, especially with compulsory administrative regulations or financial incentives. Offline interventions also had a greater effect on reducing antibiotic prescription rates than online ones. However, due to the high heterogeneity, the results should be interpreted with caution.

Our findings are consistent with previous systematic reviews worldwide [12], The results in the primary analyses indicating that educational interventions can achieve significant reductions in antibiotic prescribing, antibiotic injection rates and inappropriate prescribing, by combining with other strategies (Figure 3). As has been reported, one of the main driving factors for excessive antibiotic prescription is the inappropriateness of antibiotic use for the condition. It derives from inadequate knowledge of guideline recommendations on antibiotic indications [38], as well as habit, the use of the same antibiotics over many years [39]. Thus, comprehensive educational interventions are necessary to improve antibiotic prescription behaviors.

Though the results in subgroup analyses showed that educational interventions conducted offline seemed to be more effective than those online, it should be taken with caution since only two studies were conducted online. A meta-analysis of prescribing to children for upper respiratory infections also demonstrated the positive effect of face-to-face training for appropriate antibiotic prescribing in high-income countries (RR 0.77, 95% CI, 0.65 to 0.92) [40]. Online interventions such as sending educational text messages only may have marginal effects in reducing antibiotic prescription rates [23], while online training seemed to be effective when combined with offline educational materials [24,36]. The effect was also influenced by the types and duration of the intervention [41]. An RCT showed that short-term offline sessions failed to reduce the antibiotic prescribing rate. The authors attributed this to the short duration and limited effect of a single educational intervention [21]. This suggests that interventions should be sustained over the long term until habits are formed. Holding conferences is a widely-used educational method, which has shown promising effects in many studies as a primary component of continuing education [42]. Future studies are needed to clarify the optimal types of educational interventions in China.

Concerning the two education-only interventions, we found that the single intervention with only brief reading materials was not effective. As a previous review suggested, a single intervention has little or no effects on prescribing behavior [43]. Nevertheless, a combination of educational and other interventions is effective (Figure 4). In our study, the three strategies most often combined with educational interventions were prescription feedback, mandatory regulations and financial rewards and sanctions, respectively. Prescription feedback has been widely recommended [44] and is well validated in other studies [45,46,47]. Feedback and audit as well as comparisons of behaviors with peers can help to maximize the impact and improve the acceptability of stewardship interventions [38]. However, we found no difference in antibiotic prescription rates with or without additional prescription feedback. This may provide additional evidence for intervention models in combination with antibiotic education.

In addition, our study indicated a stronger effect on reducing antibiotic prescribing rates among educational interventions which were combined with compulsory administrative regulations or financial incentives. This suggests that a combination of educational and mandatory interventions could have a greater impact on reducing antimicrobial prescribing rates in China. In addition to the exams included in the educational interventions, other mandatory administrative regulations such as specific prescribing targets or displaying ranking information of prescribing behavior of doctors are also helpful.

This is the first review of the effect of educational interventions conducted in Chinese outpatient settings. Our work has provided an evidence base for future studies conducted in China. Nevertheless, there are limitations. Firstly, we did not include unpublished studies in the search and identification. Some of these were probably unpublished because of non-significant changes in outcomes, introducing considerable publication bias. Secondly, due to the limited number of articles, we cannot state which educational intervention is more effective. This needs to be explored in future studies. Thirdly, since interventions were grouped when conducted, we cannot obtain the effect of a single measure, for example, the automatic prescription screening system combined with other interventions in one study [28]. Finally, since outcomes were antibiotic prescribing rates measured at the facility rather than individual level, and staffing changes were not reported, the results may be biased by inclusion, at follow-up, of doctors who had not received the intervention. Thus, future studies should explore the changes in individual doctor prescribing behavior rather than just changes in the overall antimicrobial prescribing rate [48].

Our review demonstrated that education-plus interventions can significantly reduce the antibiotic prescription rate in Chinese outpatient settings. A comprehensive approach, including education, is needed to reduce antibiotic prescribing rates. Further high-quality studies are needed to identify effective interventions to improve AMS and reduce misuse of antibiotics in China.

## Figures and Tables

**Table 1:** Characteristics of included studies analyzing the effect of education-only intervention or plus other interventions.

First Author, Year	Study Design	Participants	Setting and District	Intervention Details	Target Illness	Duration of Intervention and Data Collection	Key Outcomes
Chen et al., 2003 [33]	Cluster-RCT	Clinicians and pharmacists in 7 hospitals	7 hospitals in Peking (tertiary 1 vs. 1; secondary 1 vs. 1; primary 1 vs. 2)	Lectures on rational drug use and training on international standards; A brochure on rational drug use	Not specific	Duration: 3 months. Data were collected for 5 months both before baseline and after endline	The antibiotic prescribing rate declined (intervention: from 37.7% to 33.7%; control: 38.5% to 37.0%).
Chen et al., 2007 [34]	CBA	Medical staff	Six secondary and tertiary hospitals in Zhuhai, Guangdong	Clinical guidelines, lectures, and knowledge competition on rational antibiotic use; Financial punishment; A Monitoring-Training-Plan Team Working System	URIs	Duration: 1 year. Data were collected for 3 months before three to four rounds of intervention at four-week intervals	The antibiotics prescribing rate declined from 75.3% to 31.4%.
Xie et al., 2008 [31]	CBA	Doctors, pharmacists and caregivers	Six secondary and tertiary hospitals in Shenzhen, Guangdong	Seminars on the rational use of medicines; Books and manuals related to rational drug use; Intervention program and expected targets; Feedbacks on antibiotic prescribing	Not specific	Duration: 10 months. Data were collected for 2 months both during 1 month both before baseline and after endline	The rate of antibiotic prescription significantly reduced (from 52.9% to 30.4%).
Li et al., 2013 [32]	ITS	Doctors	123 village health clinics in Qingdao, Shandong	Prescription feedback; Trainings on antibiotic use.	Not specific	Duration: 1 year. Data were collected monthly for 10 months before intervention and 12 months after intervention	A significant 0.88% decline in average antibiotic prescription rates.
Gao et al., 2013 [24]	CBA	Doctors, pharmacists and caregivers	186 township health lefts in Xinjiang Uygur Autonomous Region	Training brochures and television program on antibiotics; Financial penalties	Not specific	Duration: 2 months Data were collected for one month both during 7 months both before baseline and 5 months after endline	The antibiotic prescribing rate declined from 61% to 45% significantly.
Chen et al., 2014 [23]	Cluster-RCT	977 health workers at recruitment	100 township health lefts in Gansu province (52 vs. 48)	(1) intervention group: text messages about recommendations for the management of the infections three times a week by computers (2) control group: a traditional one-day training program	URIs	Duration: 2 months. Data were collected for 3 months (including half month before endline) and the same period one year before the trial	Antibiotic prescription rate increased (from 50% to 67%) in the control group, unchanged (68%) in the intervention group. The knowledge score increased by 16% in the intervention group, with no significant changes in the control group.
Liu et al., 2015 [26]	CBA	Doctors	8 township health lefts in Xiaolan, Guangdong	Optimize administrative structure; Set specific antibiotic targets; Training to improve antibiotic application capacity; Prescribing feedback	Not specific	Duration: 1 year. Data were collected for 1 year both during 1 year both before baseline and after endline	The rate of antibiotic prescription (from 52.9% to 30.4%) and multiple antibiotics (from 43.5% to 22.8%) reduced. The most used antibiotics were still Cephalosporins with increasing proportion. The proportion of antibiotic prescriptions for acute URI increased (from 46.7% to 56.0%).
Bao et al., 2015 [22]	ITS	Medical workers	30 tertiary hospitals and 35 secondary hospitals nationwide	A national education programs for doctors and managerial personnel; Enforcement of mandatory administrative regulations	Not specific	Duration: 1 year. Data were collected monthly in three defined segments: Segment 1: the preparation period (July 2010 to June 2011); Segment 2: the policy intervention period (July 2011 to June 2012); and Segment 3: the assessment period (July 2012 to June 2014)	Antibiotic prescription rate significantly decreased (26.4% vs. 12.9%, 1.07% decline monthly) during the intervention period.
Tang et al., 2017 [27]	Cluster-RCT	60 doctors	Qianjiang city of Hubei province, involving 20 primary care organizations	Dissemination posters and brochures with a brief introduction on health risks of excessive use of antibiotics; Feedbacks on antibiotic prescription; Display ranking information		Duration: 1 year. Data were collected for 6 months from 4 months after baseline and for 1 year before baseline	Antibiotics prescribing rate declined (intervention: from 90.7% to 86.1%; control: from 90.6% to 88.0%). No effect on reducing the overall prescribing rate of injection antibiotics (*p* > 0.05).
Wei et al., 2017 [30]	Cluster-RCT	Doctors	25 township hospitals within the rural, low-income province of Guangxi in western China	Clinical guidelines based on the latest Chinese and international antibiotic-use guidelines; 2-h interactive training session; Monthly peer-review meetings with feedbacks; Leaflets and a video about antibiotics	URIs for children aged 2–14	Duration: 6 months. Data were collected during the 3 months prior to the baseline, and during the final 3 months of endline	The antibiotics prescribing rate declined (intervention: from 82% to 40%; control: from 75% to 70%; *p* < 0.01). No difference of multiple and injection antibiotic prescription rates between the two groups was observed at endline (*p* > 0.05).
Li et al., 2017 [37]	CBA	Health workers	17 primary health lefts in Jiande, Zhejiang	Training in rational drug use; Inclusion of antibiotic use in assessment indicators; Feedback and audit of junior centre doctors’ prescriptions;	Not specific	Duration: 3 months. Data were collected for one month both during 1 month both before baseline and after endline	The rates of inappropriate antibiotic prescription (from 28.7% to 20.8%), multiple antibiotics (from 26.7% to 16.8%), and antibiotic injection (from 60.7% to 47.3%) reduced significantly. No major change in the types of antibiotics used, and cephalosporins were the most used.
Wang et al., 2019 [28]	ITS	Medical staff	Beijing Chaoyang Hospital in Peking	Clinical pharmacists trained the medical staff on rational use of antibiotics both online and offline; Program and regulations on antibiotic use; Automatic prescription screening system; Financial reward and punishment; Prescription audit and feedback	Not specific	Duration: 36 months. Data were collected monthly in three defined stages: Stage 1: baseline phase (July 2010 to June 2011); stage 2: intervention phase (July 2011 to December 2013); and stage 3: stability phase (January 2014 to December 2016)	The average antibiotic prescription rates declined 0.33% during the intervention period.
Wei et al., 2019 [29]	Cluster-RCT	Doctors	25 township hospitals within the rural, low-income province of Guangxi	Clinical guidelines based on the latest Chinese and international antibiotic-use guidelines; 2-h interactive training session; Monthly peer-review meetings with feedbacks; Leaflets and a video about antibiotics	URIs for children aged 2–14	Duration: 6 months. Data were collected for 3 months prior to baseline, the final 3 months of endline, and at 18-month follow-up (during the final 3 months of the 18-month period since the intervention was first implemented)	the antibiotics prescribing rate declined (intervention: from 84% to 54%; control: from 76% to 75%; adjusted risk difference 36%, *p* < 0.01). No difference of injection antibiotic prescription rate Reported better knowledge and confidence in qualitative study.
Fang et al., 2019 [35]	CBA	Health workers	all township (town or village) health lefts in Zhenjiang, Jiangsu	Training for medical personnel by experts (14 sessions were organized, with more than 1400 people trained); helped formulate the program of stewardship	Not specific	Duration: 1 year. Data were collected for one month both during 1 month both before baseline and after endline	The rates of total antibiotic prescription (from 26.4% to 16.9%), inappropriate antibiotic prescription (from 34.1% to 17.4%) and antibiotic injection rate (from 15.2% to 41.1%) significantly declined.
Li et al., 2020 [25]	ITS	Doctors	11 CHCs in Shenzhen, Guangdong	Educational programs for clinicians every 6 months containing a test; A system of reward and punishment; Antibiotic prescribing management and audit	Not specific	Duration: 2 years. Data were collected monthly: 24 months before the intervention (January 2010–December 2011) and 48 months after the intervention (January 2012–December 2015)	A 3.1% decline in average antibiotic prescription rate during the intervention period with a cumulative effect of 74.0% decline by the end of the study.
Jin et al., 2021 [36]	CBA	Doctors and pharmacists	5 township health lefts in Yichun, Sichuan	Higher-level hospitals form medical associations and regularly visit township health lefts to give lectures; Feedback on prescriptions through WeChat and telephone	Not specific	Duration: 1 year. Data were collected during 1 year before and 1 year after implementation of intervention	The rates of antibiotic prescription (from 51.8% to 41.2%, *p* < 0.05), inappropriate antibiotic prescription (75.3% to 52.4%, *p* < 0.05), multiple antibiotics (12.3% to 12.1%, *p* > 0.05), and antibiotic injection reduced (60.0% to 47.1%, *p* < 0.05).

Abbreviations: CBA, controlled before-after study; CHC, community health center; ITS, interrupted time series analysis; RCT, randomized control trial; URI, upper respiratory infection; WHO, World Health Organization.

## Data Availability

The datasets used and/or analyzed during the study are available from the corresponding authors on reasonable request.

## Supplementary Materials

The following supporting information can be downloaded at: https://www.mdpi.com/article/10.3390/antibiotics11060791/s1. Table S1: PRISMA checklist of the systematic reviews and meta-analysis; Table S2: Search terms and search strategies in databases; Table S3: Criteria for the inappropriateness of antibiotic prescription; Figure S1: Risk of bias graph for studies included; Figure S2: Leave one out analysis of the effect of educational interventions on antibiotic prescription rates; Figure S3: Effect of the educational intervention on the antibiotic injection rates; Figure S4: Effect of the educational intervention on the multiple antibiotic rates; Figure S5: Effect of the educational intervention on the inappropriateness of antibiotic prescription.
