# Supplementary material for: Effectiveness of Educational Interventions for Health Workers on Antibiotic Prescribing in Outpatient Settings in China: A Systematic Review and Meta-Analysis
Source: Antibiotics (Basel). 2022 Jun 10;11(6):791. doi: 10.3390/antibiotics11060791 (PMC9220158; doi:10.3390/antibiotics11060791)
Supplement: Supplementary file 1 [file antibiotics-11-00791-s001.zip › antibiotics-1717522-supplementary.pdf]

# Effectiveness of educational interventions for health workers on antibiotic prescribing in outpatient settings in China: a systematic review and meta-analysis

**Kunhua Zheng[1,†], Ying Xie[2,†], Lintao Dan[2,†], Meixian Mao[1], Jie Chen[2], Ran Li[2,3], Xuanding Wang[4,*] and Therese Hesketh[2,3]**

1 People's Hospital of Kaihua, 324300 Quzhou, China
2 Center for Global Health, Zhejiang University School of Medicine, 310000 Hangzhou, China
3 Institute for Global Health, University College London, WC1E 6BT London, UK
4 Department of Antimicrobial Stewardship, The Second Affiliated Hospital of Zhejiang University School of Medicine, 310000 Hangzhou, China.
* Correspondence: Xuanding Wang, Department of Antimicrobial Stewardship, The Second Affiliated Hospital of Zhejiang University School of Medicine, Hangzhou, China. Email: xudwang@zju.edu.cn
† Kunhua Zheng, Ying Xie and Lintao Dan contributed equally.

**Supplementary Materials**

Table S1 PRISMA checklist of the systematic reviews and meta-analysis

Table S2 Search terms and search strategies in databases

Table S3 Criteria for inappropriateness of antibiotic prescription

Figure S1 Risk of bias graph for studies included

Figure S2 Leave one out analysis of the effect of educational interventions on antibiotic prescription rates

Figure S3 Effect of the educational intervention on the antibiotic injection rates

Figure S4 Effect of the educational intervention on the multiple antibiotic rates

Figure S5 Effect of the educational intervention on the inappropriateness of antibiotic prescription

**Table S1** PRISMA checklist of the systematic reviews and meta-analysis

| Section and Topic | Item # | Checklist item | Location where item is reported |
|---|---|---|---|
| **TITLE** | | | |
| Title | 1 | Identify the report as a systematic review. | 1 |
| **ABSTRACT** | | | |
| Abstract | 2 | See the PRISMA 2020 for Abstracts checklist. | 1 |
| **INTRODUCTION** | | | |
| Rationale | 3 | Describe the rationale for the review in the context of existing knowledge. | 1-2 |
| Objectives | 4 | Provide an explicit statement of the objective(s) or question(s) the review addresses. | 2 |
| **METHODS** | | | |
| Eligibility criteria | 5 | Specify the inclusion and exclusion criteria for the review and how studies were grouped for the syntheses. | 2-3 |
| Information sources | 6 | Specify all databases, registers, websites, organisations, reference lists and other sources searched or consulted to identify studies. Specify the date when each source was last searched or consulted. | 3 |
| Search strategy | 7 | Present the full search strategies for all databases, registers and websites, including any filters and limits used. | Table S2 |
| Selection process | 8 | Specify the methods used to decide whether a study met the inclusion criteria of the review, including how many reviewers screened each record and each report retrieved, whether they worked independently, and if applicable, details of automation tools used in the process. | 3 |
| Data collection process | 9 | Specify the methods used to collect data from reports, including how many reviewers collected data from each report, whether they worked independently, any processes for obtaining or confirming data from study investigators, and if applicable, details of automation tools used in the process. | 3 |

| Section and Topic | Item # | Checklist item | Location where item is reported |
|---|---|---|---|
| Data items | 10a | List and define all outcomes for which data were sought. Specify whether all results that were compatible with each outcome domain in each study were sought (e.g. for all measures, time points, analyses), and if not, the methods used to decide which results to collect. | 3 |
| | 10b | List and define all other variables for which data were sought (e.g. participant and intervention characteristics, funding sources). Describe any assumptions made about any missing or unclear information. | 3 |
| Study risk of bias assessment | 11 | Specify the methods used to assess risk of bias in the included studies, including details of the tool(s) used, how many reviewers assessed each study and whether they worked independently, and if applicable, details of automation tools used in the process. | 4 |
| Effect measures | 12 | Specify for each outcome the effect measure(s) (e.g. risk ratio, mean difference) used in the synthesis or presentation of results. | 4 |
| Synthesis methods | 13a | Describe the processes used to decide which studies were eligible for each synthesis (e.g. tabulating the study intervention characteristics and comparing against the planned groups for each synthesis (item #5)). | 3 |
| | 13b | Describe any methods required to prepare the data for presentation or synthesis, such as handling of missing summary statistics, or data conversions. | NA |
| | 13c | Describe any methods used to tabulate or visually display results of individual studies and syntheses. | NA |
| | 13d | Describe any methods used to synthesize results and provide a rationale for the choice(s). If meta-analysis was performed, describe the model(s), method(s) to identify the presence and extent of statistical heterogeneity, and software package(s) used. | 4 |
| | 13e | Describe any methods used to explore possible causes of heterogeneity among study results (e.g. subgroup analysis, meta-regression). | 4 |

| Section and Topic | Item # | Checklist item | Location where item is reported |
|---|---|---|---|
| | 13f | Describe any sensitivity analyses conducted to assess robustness of the synthesized results. | NA |
| Reporting bias assessment | 14 | Describe any methods used to assess risk of bias due to missing results in a synthesis (arising from reporting biases). | 4 |
| Certainty assessment | 15 | Describe any methods used to assess certainty (or confidence) in the body of evidence for an outcome. | 4 |
| **RESULTS** | | | |
| Study selection | 16a | Describe the results of the search and selection process, from the number of records identified in the search to the number of studies included in the review, ideally using a flow diagram. | Figure 1 |
| | 16b | Cite studies that might appear to meet the inclusion criteria, but which were excluded, and explain why they were excluded. | NA |
| Study characteristics | 17 | Cite each included study and present its characteristics. | 4-10 |
| Risk of bias in studies | 18 | Present assessments of risk of bias for each included study. | Figure 2 |
| Results of individual studies | 19 | For all outcomes, present, for each study: (a) summary statistics for each group (where appropriate) and (b) an effect estimate and its precision (e.g. confidence/credible interval), ideally using structured tables or plots. | Table 1 |
| Results of syntheses | 20a | For each synthesis, briefly summarise the characteristics and risk of bias among contributing studies. | 10 |
| | 20b | Present results of all statistical syntheses conducted. If meta-analysis was done, present for each the summary estimate and its precision (e.g. confidence/credible interval) and measures of statistical heterogeneity. If comparing groups, describe the direction of the effect. | 12-14<br>Figure 3<br>Figure 4 |
| | 20c | Present results of all investigations of possible causes of heterogeneity among study results. | Figure 3 |
| | 20d | Present results of all sensitivity analyses conducted to assess the robustness of the synthesized results. | NA |

| Section and Topic | Item # | Checklist item | Location where item is reported |
|---|---|---|---|
| Reporting biases | 21 | Present assessments of risk of bias due to missing results (arising from reporting biases) for each synthesis assessed. | NA |
| Certainty of evidence | 22 | Present assessments of certainty (or confidence) in the body of evidence for each outcome assessed. | 10 |
| **DISCUSSION** | | | |
| Discussion | 23a | Provide a general interpretation of the results in the context of other evidence. | 15 |
| | 23b | Discuss any limitations of the evidence included in the review. | 15-16 |
| | 23c | Discuss any limitations of the review processes used. | 15-16 |
| | 23d | Discuss implications of the results for practice, policy, and future research. | 16 |
| **OTHER INFORMATION** | | | |
| Registration and protocol | 24a | Provide registration information for the review, including register name and registration number, or state that the review was not registered. | 2 |
| | 24b | Indicate where the review protocol can be accessed, or state that a protocol was not prepared. | 2 |
| | 24c | Describe and explain any amendments to information provided at registration or in the protocol. | NA |
| Support | 25 | Describe sources of financial or non-financial support for the review, and the role of the funders or sponsors in the review. | 16 |
| Competing interests | 26 | Declare any competing interests of review authors. | 16 |
| Availability of data, code and other materials | 27 | Report which of the following are publicly available and where they can be found: template data collection forms; data extracted from included studies; data used for all analyses; analytic code; any other materials used in the review. | 16 |

*From:* Page MJ, McKenzie JE, Bossuyt PM, Boutron I, Hoffmann TC, Mulrow CD, et al. The PRISMA 2020 statement: an updated guideline for reporting systematic reviews. BMJ 2021;372:n71. doi: 10.1136/bmj.n71

**Table S2** Search terms and search strategies in databases

**1. PUBMED 2021.08.03**

| **Search number** | **Query** | **Results** |
|---|---|---|
| 1 | Intervention* OR Education* OR Curriculum OR Training OR Workshop* OR Program* OR Innovation* OR Approach* OR conference* OR lecture* OR seminar* OR tutorial* OR feedback* OR reminder* OR assist* OR video* OR instruct* OR material* OR guideline* | 8,203,145 |
| 2 | Anti biotic* OR Antibiotic* OR Antimicrobial* OR Anti microbial* OR anti bacterial* OR antibacterial* OR Anti infective OR Microbicide* | 2,144,533 |
| 3 | Outpatient* OR Out-patient* OR hospital* OR clinic* OR health care* OR healthcare* OR ambulatory care* OR ambulatory health OR urgent care* | 10,873,414 |
| 4 | Prescrib* OR Prescription* | 252,230 |
| 5 | China OR Chinese | 2,284,624 |
| 6 | #1 AND #2 AND #3 AND #4 AND #5 | **574** |

**2. EMBASE 2021. 08.03**

| | | |
|---|---|---|
| 1 | Intervention* OR Education* OR Curriculum OR Training OR Workshop* OR Program* OR Innovation* OR Approach* OR conference* OR lecture* OR seminar* OR tutorial* OR feedback* OR reminder* OR assist* OR video* OR instruct* OR material* OR guideline* | 14,043,908 |
| 2 | Antibiotic* OR (Anti AND biotic*) OR Antimicrobial* OR (Anti AND microbial*) OR (anti AND bacterial*) OR antibacterial* OR (Anti AND infective) OR Microbicide* | 1,164,684 |
| 3 | Outpatient* OR 'Out patient*' OR Hospital* OR Clinic* OR (Health AND Care) OR (Ambulatory AND Care) OR (Ambulatory AND Health) OR (Urgent AND Care) | 18,751,752 |
| 4 | Prescrib* OR Prescription* | 471,600 |
| 5 | China OR Chinese | 2,763,396 |
| 6 | #1 AND #2 AND #3 AND #4 AND #5 | **823** |

**3. Web of Science 2021.08.03**

| | | |
|---|---|---|
| 1 | Intervention* OR Education* OR Curriculum OR Training OR Workshop* OR Program* OR Innovation* OR Approach* OR conference* OR lecture* OR seminar* OR tutorial* OR feedback* OR reminder* OR assist* OR video* OR instruct* OR material* OR guideline* | 33,888,931 |
| 2 | Antibiotic* OR (Anti biotic*) OR Antimicrobial* OR (Anti microbial*) OR (anti bacterial*) OR antibacterial* OR (Anti infective) OR Microbicide* | 1,872,372 |
| 3 | Outpatient* OR (Out patient*) OR Hospital* OR Clinic* OR (Health Care) OR (Ambulatory Care) OR (Ambulatory Health) OR (Urgent Care) | 16,060,019 |
| 4 | Prescrib* OR Prescription* | 540,019 |

| | | |
|---|---|---|
| 5 | China OR Chinese | 2,299,169 |
| 6 | #1 AND #2 AND #3 AND #4 AND #5 | **449** |

**4. Ovid Medline 2021.08.03**

| | | |
|---|---|---|
| 1 | Intervention* OR Education* OR Curriculum OR Training OR Workshop* OR Program* OR Innovation* OR Approach* OR conference* OR lecture* OR seminar* OR tutorial* OR feedback* OR reminder* OR assist* OR video* OR instruct* OR material* OR guideline* | 7005811 |
| 2 | Antibiotic* OR (Anti biotic*) OR Antimicrobial* OR (Anti microbial*) OR (anti bacterial*) OR antibacterial* OR (Anti infective) OR Microbicide* | 760963 |
| 3 | Outpatient* OR (Out patient*)OR Hospital* OR Clinic* OR (Health Care) OR (Ambulatory Care) OR (Ambulatory Health) OR (Urgent Care) | 6851703 |
| 4 | Prescrib* OR Prescription* | 250966 |
| 5 | China OR Chinese | 502208 |
| 6 | #1 AND #2 AND #3 AND #4 AND #5 | **217** |

Note: We included related terms and use advanced search.

**5. Cochrane Library 2021.08.03**

| | | |
|---|---|---|
| 1 | Intervention* OR Education* OR Curriculum OR Training OR Workshop* OR Program* OR Innovation* OR Approach* OR conference* OR lecture* OR seminar* OR tutorial* OR feedback* OR reminder* OR assist* OR video* OR instruct* OR material* OR guideline* | 852257 |
| 2 | Antibiotic* OR (Anti biotic*) OR Antimicrobial* OR (Anti microbial*) OR (anti bacterial*) OR antibacterial* OR (Anti infective) OR Microbicide* | 52588 |
| 3 | Outpatient* OR (Out patient*) OR Hospital* OR Clinic* OR (Health Care) OR (Ambulatory Care) OR (Ambulatory Health) OR (Urgent Care) | 1366508 |
| 4 | Prescrib* OR Prescription* | 39827 |
| 5 | China OR Chinese | 96019 |
| 6 | #1 AND #2 AND #3 AND #4 AND #5 | **286** |

Note: We used “All Text” and only selected trials and finally included 59 items.

**6. China National Knowledge Infrastructure, CNKI 2021.08.03**

| | | |
|---|---|---|
| 1 | SU= ‘Intervention’ + ‘Education’ + ‘Course’ + ‘Lecture’ + ‘Training’ + ‘Training’ + ‘Feedback’ + ‘Review’ | 11,800,113 |
| 2 | SU= ‘Outpatient’ + ‘Ambulatory’ + ‘Community’ + ‘Hospital’ + ‘Emergency’ + ‘Clinic’, | 54,438 |
| 3 | SU= ‘Antibiotic’ + ‘Antimicrobial Resistant’ + ‘Resistant’ + ‘Antimicrobial’ | 1,859 |
| 4 | SU= ‘Prescription’ | 1,405 |

Note: Search in the results and only included journal literature. (search terms in Chinese)

**7. Wanfang Database 2021. 08.03**

| | | |
|---|---|---|
| 1 | Intervention OR Education OR Course OR Lecture OR Training OR Training OR Feedback OR Review | 931,660 |
| 2 | Outpatient OR Ambulatory OR Community OR Hospital OR Emergency OR Clinic | 561,493 |
| 3 | Antibiotic OR Antimicrobial Resistant OR Resistant OR Antimicrobial | 340,898 |
| 4 | Prescription | 87,883 |
| 5 | #1 AND #2 AND #3 AND #4 | **1,695** |

Note: Professional search + journal articles + subject search + (Chinese and English expansion + subject term expansion) (search terms in Chinese)

**8. China Science and Technology Journal Database, SINOMED 08.03**

| | | |
|---|---|---|
| 1 | Intervention OR Education OR Course OR Lecture OR Training OR Training OR Feedback OR Review | 585,325 |
| 2 | Outpatient OR Ambulatory OR Community OR Hospital OR Emergency OR Clinic | 590,251 |
| 3 | Antibiotic OR Antimicrobial Resistant OR Resistant OR Antimicrobial | 972,049 |
| 4 | Prescription | 188,914 |
| 5 | #1 AND #2 AND #3 AND #4 AND #5 | **1,481** |

Note: (search terms in Chinese)

**Table S3** Criteria for inappropriateness of antibiotic prescription

| | Criteria |
|---|---|
| Jin et al. 2021 | (1) Inappropriate indications; (2) inappropriate drug selection; (3) inappropriate usage; (4) unsuitable dosage; (5) inappropriate combined medication; (6) irregular prescription writing |
| Liu et al. 2015 | (1) Inappropriate indications; (2) inappropriate drug selection; (3) inappropriate usage; (4) unsuitable dosage; (5) inappropriate combined medication |
| Li et al. 2017 | (1) Inappropriate indications; (2) inappropriate drug selection; (3) inappropriate usage; (4) inappropriate combined medication |
| Fang et al. 2019 | Unspecified |

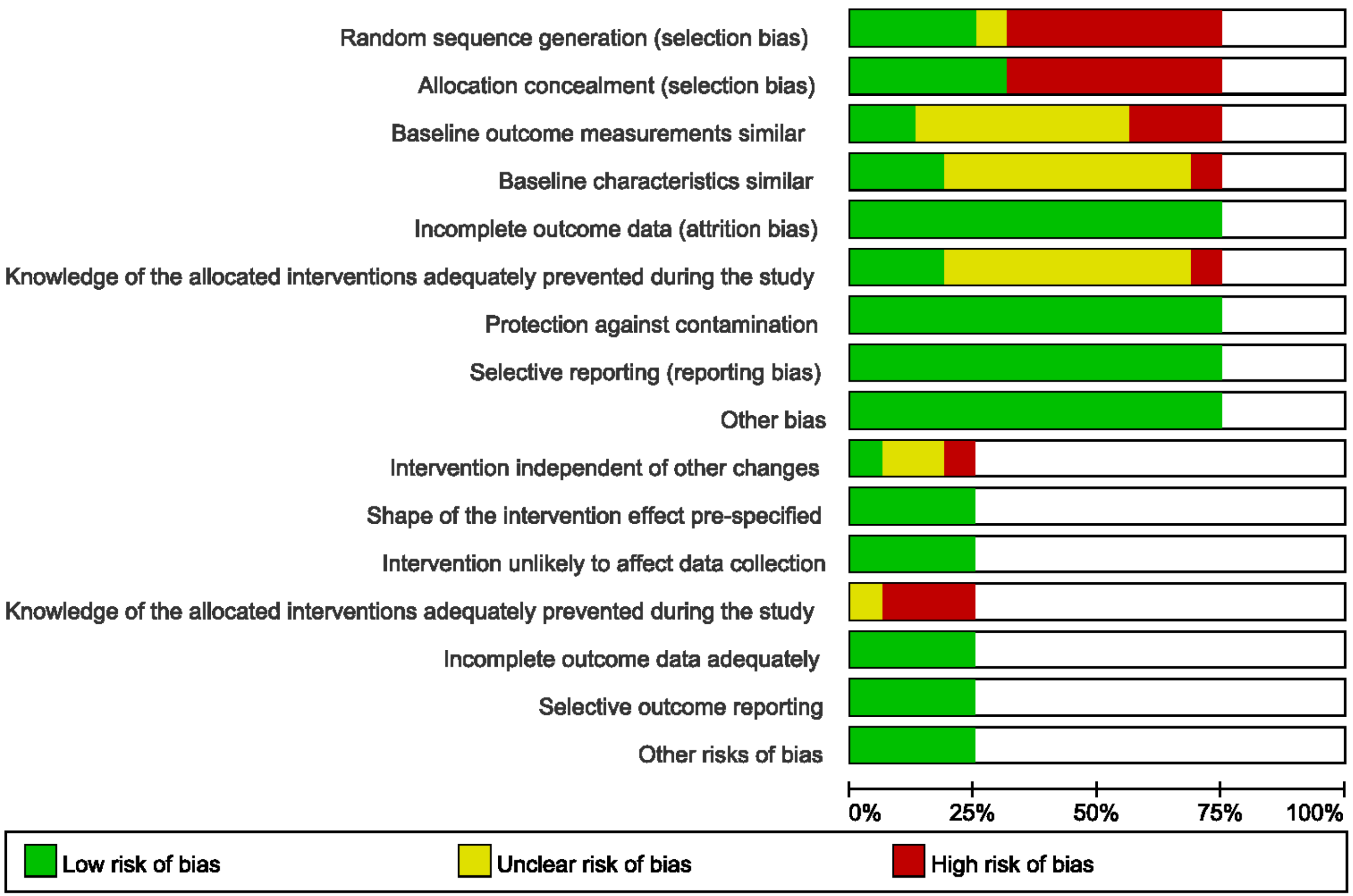


**Figure S1** Risk of bias graph for studies included

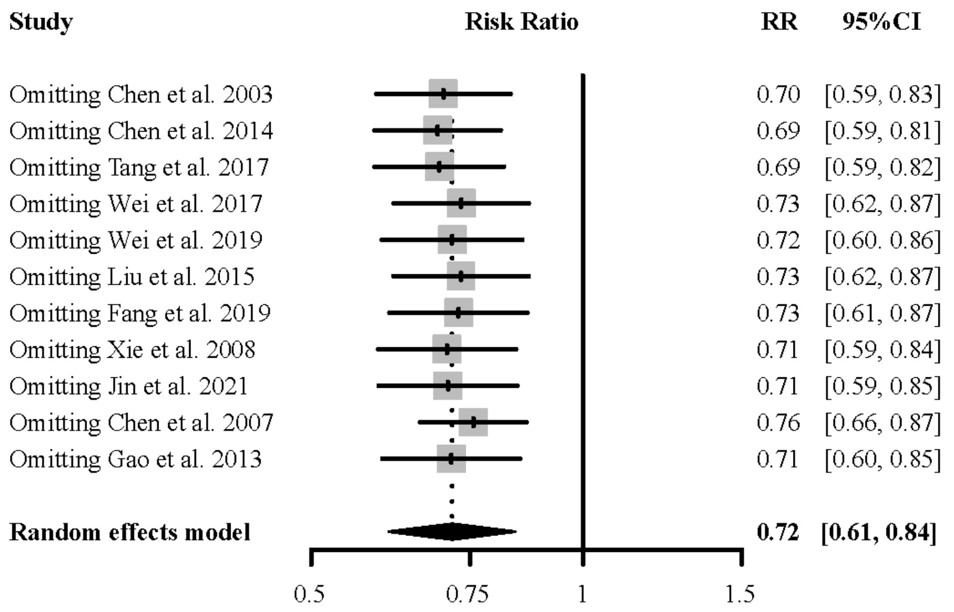


**Figure S2** Leave one out analysis of the effect of educational interventions on antibiotic prescription rates

| Study | Intervention Events | Intervention Total | Control Events | Control Total | RR | 95%CI | Weight |
|---|---|---|---|---|---|---|---|
| Chen et al. 2007 | 84 | 300 | 454 | 1054 | 0.65 | [0.54, 0.79] | 11.8% |
| Li et al. 2017 | 804 | 1700 | 1032 | 1700 | 0.78 | [0.73, 0.83] | 16.7% |
| Tang et al. 2017 | 11924 | 17021 | 11615 | 14937 | 0.90 | [0.89, 0.91] | 17.5% |
| Wei et al. 2017 | 386 | 943 | 785 | 1782 | 0.93 | [0.85, 1.02] | 15.8% |
| Fang et al. 2019 | 128 | 599 | 275 | 1426 | 1.11 | [0.92, 1.34] | 12.1% |
| Wei et al. 2019 | 266 | 2748 | 365 | 2772 | 0.74 | [0.63, 0.85] | 13.6% |
| Jin et al. 2021 | 97 | 206 | 155 | 259 | 0.79 | [0.66, 0.94] | 12.5% |
| **Random effects model** | | **23517** | | **23930** | **0.83** | **[0.74, 0.94]** | **100.0%** |

Heterogeneity: $I^2 = 86\%$, $\tau^2 = 0.0199$, $p < 0.01$

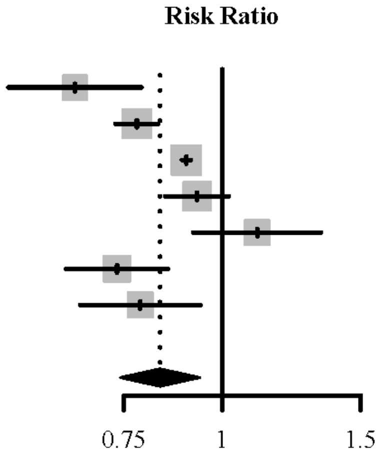


**Figure S3** Effect of the educational intervention on the antibiotic injection rates

| Study | Intervention Events | Intervention Total | Control Events | Control Total | RR | 95%CI | Weight |
|---|---|---|---|---|---|---|---|
| Li et al. 2017 | 286 | 1700 | 454 | 1700 | 0.63 | [0.55, 0.72] | 20.8% |
| Tang et al. 2017 | 2582 | 17021 | 2373 | 14937 | 0.95 | [0.91. 1.01] | 21.0% |
| Wei et al. 2017 | 87 | 943 | 117 | 1782 | 1.41 | [1.08, 1.83] | 20.1% |
| Wei et al. 2019 | 54 | 2748 | 209 | 2772 | 0.26 | [0.19, 0.35] | 19.9% |
| Jin et al. 2021 | 25 | 206 | 32 | 259 | 0.98 | [0.60, 1.60] | 18.2% |
| **Random effects model** | | **22618** | | **21450** | **0.73** | **[0.42, 1.29]** | **100.0%** |

Heterogeneity: $I^2 = 96\%$, $\tau^2 = 0.3947$, $p < 0.01$

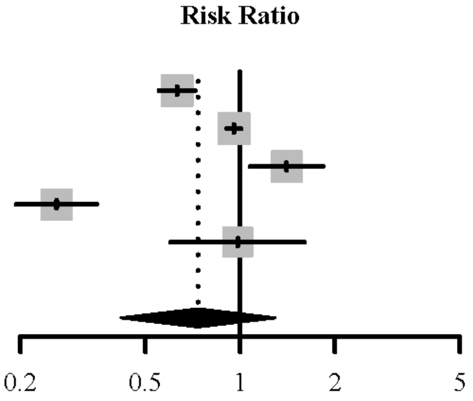


**Figure S4** Effect of the educational intervention on the multiple antibiotic rates

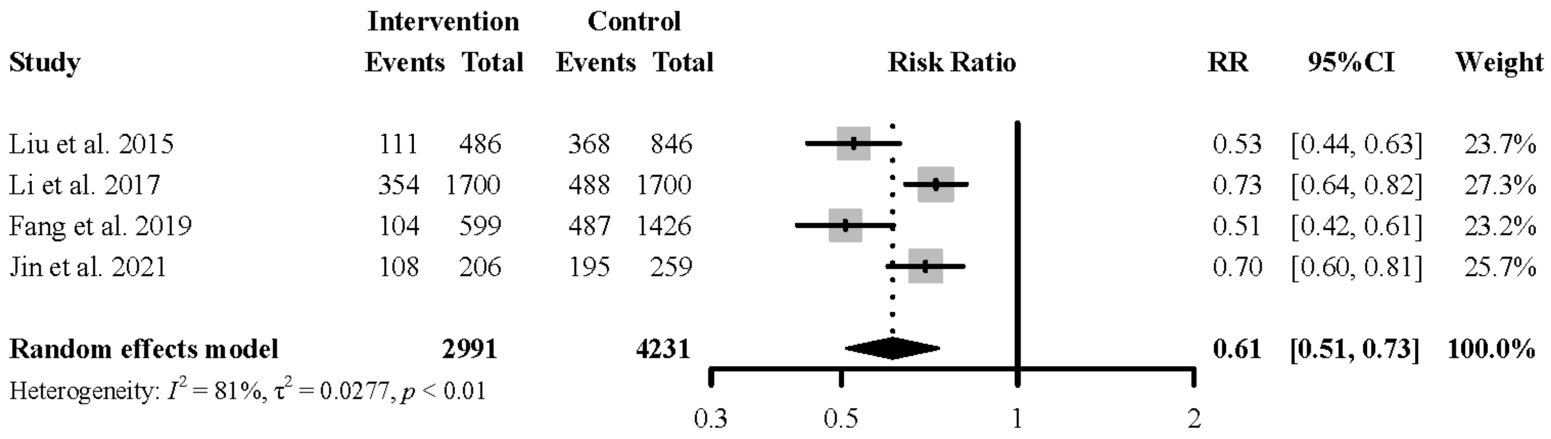


**Figure S5** Effect of the educational intervention on the inappropriateness of antibiotic prescription
